# A Single Chain Fragment Variant Binding Misfolded Alpha-Synuclein Exhibits Neuroprotective and Antigen-Specific Anti-Inflammatory Properties

**DOI:** 10.3390/cells11233822

**Published:** 2022-11-29

**Authors:** Michael Fassler, Clara Benaim, Jacob George

**Affiliations:** 1 Heart Center, Kaplan Medical Center, Rehovot 76100, Israel; 2 Cognyxx Ltd., Tel Aviv 6578317, Israel

**Keywords:** synucleinopathies, immunotherapy, alpha synuclein, neurodegeneration, nose to brain delivery

## Abstract

Introduction. Alpha synuclein (αSyn) misfolding plays a requisite role in the pathogenesis of synucleinopathies. Direct toxicity to neurons, triggering neuroinflammation as well as the spreading and seeding of αSyn pathology are essential pathogenetic underlying mechanisms. Immunotherapy in experimental Parkinson’s disease (PD) has been shown to be consistently effective in preclinical models, yet the initial clinical trials with monoclonal antibodies (mAbs) yielded marginal results if any. Aiming to overcome some of the limitation of this approach, we aimed to select an αSyn binding scFv antibody format and test it in multiple experimental PD in vivo models. Methods. We cloned the lead αSyn scFv based on preselection of human phage display libraries of human Fab. The selected of scFv targeting both oligomers and pre-formed fibrils (PFF) of αSyn were tested for their ability to protect neurons from triggered toxicity, influence their uptake to microglia, and accelerate misfolded αSyn degradation. The lead scFv- sMB08, was also tested for its ability to impact αSyn aggregation as well as spreading and seeding. Results. sMB08 was shown to protect neurons from misfolded αSyn mediated toxicity, promote its intracellular degradation, and to reduce its uptake by microglia. sMB08 exhibited anti-inflammatory properties, including its ability to attenuate adaptive αSyn autoimmunity and ameliorate proinflammatory cytokine expression in brains of mice stereotactically injected with PFF. Employing three experimental models of PD, intranasal treatment with sMB08 attenuated motoric dysfunction and achieved acceptable brain levels by pharmacokinetic analysis, leading to significant preservation of dopaminergic n neurons. Conclusion: sMB08, a scFv targeting both αSyn oligomers and PFF, due to its small size facilitating paraneural brain penetration and avoidance of nonspecific inflammation, appears as an attractive approach to test in patients with PD by addressing the major mechanisms that mediate misfolded αSyn driven pathology.

## 1. Introduction

Alpha synucleinopathies comprise a group of neurodegenerative disorders, including PD, multisystem atrophy, and dementia with Lewy bodies [1]. Other than the common denominator of the three which encompasses a varying mix of different movement disorders and cognitive dysfunction, there appears to be a shared triggering pathological expression of misfolded alpha synuclein. This 14-kd soluble monomeric protein appears to play an important role in the functional state of the neuron and comprises 0.5–1% of the total brain’s soluble fraction [2,3].

Multiple factors appear to mediate the propensity of αSyn to undergo misfolding and subsequent aggregation until assuming the mature forms known as Lewy bodies [1,2,3]. These steps include the formation of small oligomers and low molecular aggregates as well as PFFs and subsequently, mature aggregates.

The pathogenesis of synucleinopathies is complex and multifactorial and likely involves direct triggering of neurotoxicity and neurodegeneration by αSyn oligomers and PFF, that also activate the innate immune system to result in neuroinflammation [4]. These processes tend to ignite each other creating a vicious cycle that further perpetuates neuronal loss. In this respect, it is also important to conceptualize the potential importance of spreading and seeding of the pathology that likely mediates the progression of the disease [5]. The exteriorized misfolded αSyn that is being uptaken by surrounding neurons serves as a seed leading to corruption of endogenous αSyn and thus further propagates the pathology along nerves from distal locations like the gastrointestinal tract [6]. Although no approved disease modifying agents for either synucleinopathies are available, considerable research is directed at targeting the essential mechanisms mediating misfolded αSyn induced pathology [7,8,9]. Indeed, protecting against toxicity is attempted by attenuating αSyn aggregation and constraining neuroinflammation. The concept of using mAbs to treat neurodegenerative diseases has been intensely studied in multiple preclinical and clinical studies in patients with Alzheimer’s disease [10]. The idea is to target extracellular beta amyloid and facilitate Fc-gamma mediated clearance of beta amyloid via microglia, thereby reducing plaque burden. This has been successful in preclinical trials whereas in humans it is clear at this point that target engagement, namely a reduction in plaque size, is achievable despite mild if any impact on cognition [11]. These studies led to interest in applying mAbs targeting misfolded forms of αSyn in PD based on the conception that modified toxic forms of the protein are also available extracellularly, so that they can be targeted by the large non cell penetrating mAbs [12,13]. Several preclinical studies, predominantly in mice, lent support to this hypothesis, showing that mAbs targeting various forms of misfolded αSyn led to the attenuation of αSyn pathology as well as motoric function in inducible and genetic models of PD [11]. However, two mAbs that recently completed phase II studies in patients with PD, despite having achieved target engagement, had either neutral or mildly positive effects on the clinical endpoints [14,15,16]. The reason for the disappointing results despite effective target engagement could be related to the observation that the majority of the misfolded αSyn pool resides in the intracellular rather than the extracellular space that is not available for targeting by full length mAbs [6].

Single chain variable fragment (scFv), with one sixth the molecular weight of full length mAb, possesses the VH and VL regions combined by a short linker [17]. scFvs are the smallest mAb fragments that still can retain a good affinity to the antigen. Recently a misfolded αSyn binding scFv was designed that was linked to a cargo peptide and tested for its ability to attenuate toxicity, aggregation and spreading of αSyn in vitro [18,19]. In vivo studies usually necessitate driving expression of the scFv by using viruses that will infect the neurons, a complex and potentially dangerous approach with unpredictable pharmacodynamics. Herein, we have made an elaborate screening of a fully human Fab display library from which we cloned the chosen αSyn binding scFv. The selected scFv targeted both oligomeric and PFF αSyn forms, protected neurons from misfolded αSyn toxicity, spreading and seeding, and innate/adaptive immunity. Intranasal delivery of the scFv led to a significant attenuation of motoric dysfunction in three preclinical models of PD with different etiologies.

## 2. Materials and Methods

### 2.1. Generation of scFv and scFv-Fc from Fab Display Libraries of Healthy Volunteers

A human Fab library with a repertoire from over 110 individuals and diversity of 1 × 10^11^ was screened against recombinant modified human αSyn (PFF and oligomers). The use of this library allows for the identification of combinations of variable heavy and variable light chains that are not found together in any of the 110 individuals. High-affinity binders were selected by 3 rounds of bio-panning in which positive clones were identified and sequenced. After additional selection, a standard direct ELISA was performed using mouse or human αSyn PFFs (1 µg/mL) as the coating protein (100 mM bicarbonate/carbonate coating buffer) for the top 3 high binders. After two hours of incubation with blocking buffer (1% BSA/PBS), serial dilutions (1:2) of sMB08-Fc, sMB10-Fc, and sMB12-Fc antibodies in 0.1% BSA/TBST buffer were added to each well and incubated for 1 h at room temperature with gentle shaking. This was followed by secondary antibody goat anti human conjugated to HRP (0.1% BSA/TBST buffer) and incubation for an additional 1 h at room temperature with gentle shaking before measuring absorbent in a microplate reader (Tecan, Männedorf, Switzerland) at 450 nm.

### 2.2. Generation of scFv-Fc/scFv

sMB08, sMB10 and sMB12 were cloned and expressed as scFv with a molecular weight of 25 KDa. The GSSSSx3 linker was used to link the variable regions from heavy (VH) and light (VL) chains. Additionally, clones were cloned to a mammalian expression vector as a scFv conjugated to human IgG1 Fc format (scFv-Fc).

### 2.3. Preparation of Alpha-Synuclein PFFs

Purified αSyn monomers (1 mg Alexotech, Umeå, Sweden) were thawed in a 1.5-mL microcentrifuge tube in a 37 °C water bath until complete thawing. The tube was centrifuged at 100,000× *g* at 4 °C for 60 min to pellet any aggregated material. Supernatant was removed and diluted with PBS in a new, low binding, sterile 1.5-mL micro centrifuge tube (Protein LoBind Tube 1.5, Eppendorf tubes, Cat no.: 022431081) for the use of generating PFFs into a final volume of 200 μL and a final concentration of 5 mg/mL. Tube was placed in a closed thermomixer (QSR Technologies, Burlington, MA, USA) at 37 °C incubation for 7d at 1000 rpm (solution should appear turbid). The solution was divided into aliquots (10–20 μL) into sterile microcentrifuge tubes and stored at −80 °C. Before use, PFF was centrifuged at 100,000× *g*, 4 °C for 60 min. The supernatant was removed and PFF was sonicated in new sterile PBS (20% power 1 s on 1 s off in 60 s total on (30 s net on time)).

### 2.4. Conjugation of PFFs and sMB08 with Alexa Fluor 488 or Cy5

PFF were labeled with Alexa Fluor 488 (Alexa Fluor^®^ 488 Labeling Kit Lightning-Link, Cat no.: ab236553) and sMB08 with Cy5 (Cy5^®^ Labeling Kit Lightning-Link, cat no.: ab188288) respectively as described in the manufacturer protocol. All materials and reagents were equilibrated to room temperature prior to use. Labeling kit modifier reagent was added (10% *v*/*v*) to the PFFs/sMB08 to be labeled, mixed gently, and incubated for 15 min. Prepared protein mixture was added to the Alexa Fluor 488/Cy5 conjugation lyophilized mix, resuspended gently, and incubated for 15 min or longer in the dark (20–25 °C). Quencher reagent (10% *v*/*v*) was added to the conjugated protein to deactivate the excess unbound label post reaction.

### 2.5. Preparation of Aldehyde Induced αSyn Oligomers

Purified αSyn monomer (2 mg/mL Alexotech, Umeå, Sweden) (in PBS, pH 7.4) was mixed together with HNE (4-Hydroxynonenal) 20 mg/mL (in DMSO) in a low binding tube (Protein LoBind Tube 1.5, Eppendorf tubes, Cat no.: 022431081) for a final concentration of 140 µM αSyn and 4200 µM HNE, respectively. Samples were incubated at 37 °C for 18 h and analyzed in SDS-PAGE, silver stain (manufacture’s protocol). A 14 KDa form is the oligomer (αSyn adopts extended conformation oligomeric form (spherical monomers to hexamers), with dimers being the major species (SDS resistant) and PFF fibril forms (dimers and above). Of note, there can be an overlap in the size but not in the structure (fibril vs. round oligomers).

### 2.6. Preparation and Purification of Mouse αSyn

Mouse αSyn gene was cloned into a mammalian expression vector with his-tag. HEK293T cells were transfected with the vector to express mouse alpha-synuclein his-tag protein and then purified in His-trap column (FPLC).

### 2.7. Purification of Human αSyn PFF from Human Brain Samples with PD

A chunk of frozen human tissue (approximately 0.5 g in weight) was rapidly minced into small fragments (approximately 1 mm^2^) on ice using a small petri-dish and a sterile scalpel blade. Minced tissue was collected in a 15 mL tube and 10 volumes of ice-cold 1XTBS buffer with protease and phosphatase inhibitors (TBSX1/1% TX-100/TBS) was added to the tube. The sample was homogenized using a mechanical homogenizer at 20,000 rpm for 10 s and cooled on ice for additional 2 min before steps were repeated three additional times. Centrifugation was performed at 1000× *g* for 5 min at 4 °C and the pellet of non-homogenized material was discarded, while the supernatant (crude homogenate) was loaded to a polycarbonate tube for ultra-centrifugation at 100,000× *g* for 1 h at 4 °C. Pellet was washed two times in five volumes of 1X TBS buffer by centrifugation at 100,000× *g* for 15 min at 4 °C each time. Final pellet was sonicated for 10 s at 20 KHz in approximately 5 volumes of 1X TBS-SDS at RT followed by ultracentrifugation at 100,000× *g* for 30 min at 25 °C and the final pellet was solubilized in 50 μL of 1X TBS-SDS-UREA buffer using a sonicator set at 20 KHz for 10 s to attain complete solubilization. This is termed the urea soluble fraction.

### 2.8. Human Brain Samples

Control and Parkinson’s disease human brain samples were supplied by the Cambridge Brain Bank supported by the NIH Cambridge Biomedical Research Centre.

### 2.9. Cell Culture

Human neuroblastoma SH-SY5Y cells were obtained via American type culture collection (ATCC; CRL-2266), cultured in Eagle’s Minimum Essential Medium (EMEM) and F12 Medium (1:1) supplemented with 10% fetal bovine serum (FBS) and 1% penicillin/streptomycin (P/S). BV-2 mouse microglia cells were obtained via Interlab cell line collection (ICLC; ATL03001) and cultured in high glucose Dulbocco’s modified Eagle’s medium (DMEM) supplemented with 10% FBS and 1% P/S. Cells were maintained in a humidified incubator with 95% air and 5% CO_2_ atmosphere at 37 °C.

### 2.10. Cell Viability Assay

The toxicity of αSyn fibrils was assessed by the MTT cell viability assay on differentiated human neuroblastoma SH-SY5Y cells. For the MTT assay, cells were plated at a density of 10,000 cells per well on 96-well plates in 100 μL of culture medium per well. Following 24 h, the medium was exchanged with 100 μL of fresh medium containing 10 μM of retinoic acid (RA) for neuronal differentiation. After 96 additional hours, 0.0625 μM of αSyn fibrils, 0.0156 μM αSyn oligomers, or human PD brain extracts were incubated with sMB08 or sham scFv (80R a proprietary unspecific protein engineered clone) for additional 4 h at room temperature and added to the differentiated neuronal cells for 24 h at 37 °C. Control samples were prepared with the addition of identical volumes of buffer. After 24 h of incubation, the cells were incubated for another 4 h with 100 μL of serum-free Dulbecco’s modified Eagle’s medium without phenol red, containing 0.5 mg/ml MTT. Then, 100 μL of cell lysis solution (20% SDS, 50% N,N-dimethylformamide) were added to each well, and the samples were incubated overnight at 37 °C to allow complete lysis. The absorbance of the formazan was measured at 570 nm in a Tecan infinite 200 pro microplate reader (Tecan, Männedorf, Switzerland).

### 2.11. Uptake of αSyn PFF and Oligomers in Cells

Human PBMCs, BV-2 (mouse microglia, ICLC ATL03001), and peritoneal mouse macrophages were split into 12 well culture plates (Greiner CELLSTAR multiwell culture plates) on the day before the experiment. 0.3 μM αSyn PFF or oligomers conjugated with Alexa 488, pre-incubated with either 0.1, 0.5, 1.5, 3, 4 and 5 μg/mL sMB08 or sMB08-Fc for 30 min at room temperature, were added to cells with culture media (RPMI 1640, 10% FBS, 2mM L-Glutamine, 1% Pen/Strep). The conditioned medium was also pre-incubated with sMB08 (without PFF/oligomers) for 30 min as control. Cells were then incubated at 37 °C for 24 h and harvested. Flow cytometry (FL1—blue laser (488 nm) was used to measure PFF/oligomers (conjugated to ALEXA fluor 488) uptake indicated as relative geomean fluorescence intensity (gMFI). Cytochalasin D (Cyto D) antibiotic was used to inhibit cellular uptake (control).

### 2.12. Aggregation Studies of αSyn Using Thioflavin T (ThT) Fluorescence

αSyn in 10 mM phosphate buffer, pH 7.4, was filtered to ensure removal of preformed aggregates and then incubated at 37 °C with shaking at 1000 rpm (Thermomixer, QSR technologies, Burlington, MA, USA) for 6 days in the presence of 2 different sMB08 concentrations. Aliquots of the reactions were removed at each time point and added to a 10 μM Thioflavin T (ThT) (Sigma, T3516, Darmstadt, Germany) solution (prepared from a stock dissolved in 10 mM phosphate buffer, pH 7.4) in a low-binding black 96-well plate (Corning). Fluorescent measurements were performed using a Tecan infinite 200 pro microplate fluorescent reader (Tecan, Männedorf, Switzerland) recording fluorescence measurements from 440 to 485 nm. All measurements were performed in triplicate.

### 2.13. Spreading and Seeding Determination of Human PFF In Vitro

Aliquots of 5 mg/mL PFF were thawed at room temperature immediately before use. PFFs were added to sterile PBS to a final concentration of 0.1 mg/mL (minimal volume for use is 200 μL), then sonicated with 60 pulses at 10% power (total of 30 s, 0.5 s on, 0.5 s off). Sonicated PFFs or equivalent volume of PBS as a control were diluted with prewarmed neuronal medium in a 1:20 dilution (with and without sMB08 treatment (15 μg/mL)) and added to neurons (primary neurons derived from wild-type, nontransgenic p0 mouse pups, [20]) 7 days after plating for additional 7–10 days. Approximately 50% of the medium was changed once a week. Next, primary neuronal cells were rinsed twice with PBS and each well was completely aspirated before adding ice cold RIPA lysis buffer with protease and phosphatase inhibitors. Neuronal cells were taken out from wells using a cell scraper and placed in a low binding 1.5 mL tubes on ice. Next, cells were sonicated (1 s on, 1 s off, time 5 s—20%) and incubated on ice for 30 min. Samples were centrifuged at 17,000× *g* at 4 °C for 60 min, and supernatants were removed to a new low-binding 1.5 mL tubes, retained on ice or in a −20 °C freezer while additional (same volume) ice-cold RIPA lysis buffer with protease and phosphatase inhibitors was added to the pellet. The pellet tube was sonicated (1 s on, 1 s off, time 5 s—20%) and centrifuged at 17,000× *g* at room temperature for additional 80 min, supernatant was discarded, and 2% (*w*/*v*) of SDS/RIPA added to the pellet with protease and phosphatase inhibitors. Cells were sonicated (1 s on, 1 s off, time 16 s—20%), and a BCA/protein assay was performed on TX-100 supernatant and SDS extract. Samples were diluted with 2% (*w*/*v*) SDS extract into Laemmli buffer 2× + beta mercaptoethanol and heated at 95 °C for 5 min before loading on a 10% (*w*/*v*) gel. Membrane was blocked for 1 h with TBST/5% (*w*/*v*) milk, stained with diluted primary antibodies mouse anti-human αSyn (clone Syn204, BioLegend, San Diego, CA, USA) 1:1000, rabbit anti-rodent αSyn (D37A6, CellSignal, Danvers, MA, USA) 1:1000, anti-GAPDH (clone 6C5, Millipore, Burlington, MA, USA) 1:2000 followed by secondary antibodies peroxidase-AffiniPure goat anti-mouse IgG (Jackson) 1:10,000, mouse anti-rabbit IgG (Jackson)) 1:10000 TBS/1% (*w*/*v*) BSA, incubated for additional 1 h (both primary and secondary separately) while shaking and rinsed × 3 times (5 min each rinse) before enhanced chemiluminescence development.

### 2.14. Pull-Down Assay (Immunoprecipitation) of Serum Derived αSyn by sMB08

Human sera and human cerebral spinal fluid (CSF) obtained from PD patients were immunoprecipitated with different amounts of sMB08-Fc (5, 10, and 50 µg) bound to Dynabeads protein A (Dynabeads^®^ Protein A for IP 1 mL, Life technologies, Grand Island, NY, USA) following manufacturer’s instructions, then separated by SDS–PAGE (Bis-Tris 4–12% acrylamide) and transferred to protran nitrocellulose membranes. Membranes were analyzed by immunoblot for αSyn using mouse anti-human αSyn (clone Syn204, BioLegend-BLG838201; 1:1000) and mouse anti-αSyn phosphor (Ser129) (clone P-syn/81A, BioLegend-BLG825701; 1:500) following peroxidase-AffiniPure goat anti-mouse IgG (Jackson; 1:10,000) before enhanced chemiluminescence development. Further, 100 ng of recombinant human αSyn PFF and PBS were immunoprecipitated with 50 µg of sMB08-Fc bound to Dynabeads as positive and negative control respectively.

### 2.15. Isolation and Culture of Human Peripheral Blood Mononuclear Cells (PBMC)

Whole blood was diluted 1:1 ratio with PBS (Ca/Mg free), gently laid in ficoll (ratio 1:2), and centrifuged for 30 min, 1500 rpm (with slow acceleration and de-acceleration (0)). Buffy coat was collected into a new tube, washed with PBS, and centrifuged for 15 min in 1200 rpm. Additional wash with PBS was supplemented, and blood was centrifuged for 15 min in 1200 rpm before cells were resuspended in 10 mL PBS for counting.

### 2.16. ELISA for Assessment of Neuroinflammatory Markers

Interleukin 6 (IL-6) and tumor necrosis factor alpha (TNFa) protein levels in cells were detected using DuoSet Elisa assay (R&D System) following manufacturer protocol.

### 2.17. ELISPOT

The assay was performed by culturing PBMCs for 14 days (dilution of 1:1 with medium supplemented with Interleukin 2 (IL-2) each couple of days) with PFF/oligomers in order to stimulate helper and cytotoxic T cells and then to test whether αSyn pff/oligomers were recognized by those cells. Specifically, culturing of PBMCs for in vitro expansion was performed by incubating in RPMI (Biological Industries, Kibbutz Beit-Haemek Israel) supplemented with 5% human AB serum (Biological Industries, Kibbutz Beit-Haemek, Israel), GlutaMAX (Gibco), and penicillin and streptomycin (Biological Industries, Kibbutz Beit-Haemek Israel) at 2 × 10^6^ per ml in the presence of αSyn PFF/oligomers at 10 μg/mL. Every three days, 10 U/mL IL-2 in medium was added to the cultures. After 14 days, cultures were stimulated with 25 μg/mL αSyn PFF or 25 μg/mL αSyn oligomers and the response was measured by interferon gamma (IFNγ) and interleukin 5 (IL-5) ELISPOT. ELISPOT antibodies, mouse anti-human IFNγ (clone 1-D1K), mouse anti-human IL-5 (clone TRFK5), mouse anti-human IFNγ–HRP (clone 7-B6-1), and mouse anti-human IL-5 biotinylated (clone 5A10) were all from Mabtech.

### 2.18. Real Time PCR for Assessment of Inflammatory Cytokine Expression

Ten-week old mice were injected with human αSyn PFF or oligomers into the striatum (with and without sMB08 treatment) to examine in vivo neuroinflammation. 72 h post-intrastriatal injections, animals were sacrificed, and brains were dissected and homogenized. RNA from whole brain and suspension cells was isolated using the SV total RNA isolation system kit (Promega) or EZ-RNA Total RNA Isolation Kit (Biological Industries, Kibbutz Beit-Haemek, Israel) respectively following the manufacturer’s protocol. Complementary DNA (cDNA) synthesis was carried out with qScript cDNA Synthesis Kit (Quanta bio) using 2 µg of total RNA as template. In order to examine neuroinflammation relative quantitation of gene expression was conducted by real time PCR carried out using TaqMan^®^ Gene Expression Assay (Applied Biosystems, Bedford, MA, USA). Fluorescent (FAM)-labeled IL-6, TNFα, INFγ, IL-12, Caspase 3, Arg1, Chi3l3, and Mrc1 were normalized to an internal control, GAPDH. All comparisons refer to sham. Analysis was performed using the comparative Ct method (ΔΔCT).

### 2.19. αSyn PFF Degradation in sMB08 Treated SH-SY5Y Cells

Human neuroblastoma SH-SY5Y cells were seeded in a 12 well tissue culture plate (3 × 10^5^/well) using 0.5 mL medium per well (MEM:DMEM F12 (1:1), 15%FBS, 4 mM L-glutamine, 0.1 mg/mL Penicillin-Streptomycin). Four hours later, the cells were treated with retinoic acid for neuronal differentiation. After three days, fresh medium with 0.2 µM PFF-488 was added to the differentiated cells followed by overnight incubation at 37 °C in 5% CO_2_ atmosphere. Sixteen (16) hours after, cells were washed with PBS and fresh medium with different concentrations of Ab sMB08 (1, 2, 3, or 4 µg/mL) was added into the cells followed by incubation at 37 °C in 5% CO_2_ for 48 h. Cells were detached from tissue culture plates using trypsin and washed twice with PBS. Subsequently, cells were quenched using trypan blue 0.4% pH = 5. Rapidly, cells were washed three times with PBS and the samples were analyzed using flow cytometry (FL1—blue laser (488 nm); CytoFLEX, Beckman Coulter) in order to measure αSyn PFF (conjugated to ALEXA fluor 488) uptake indicated as relative geomean fluorescence intensity (gMFI).

### 2.20. Animals

WT mice (C57BL/6JRccHsd background) and Rats (Sprague-Dawley) were purchased from Envigo RMS (Israel), Ltd. All housing, breeding, and procedures were performed according to the National Institute of Health (NIH) Guide for the Care and Use of Experimental Animals and approved by the Kaplan Medical Center Institutional Animal Care and Use Committee and in compliance with “The Israel Animal Welfare Act” under local protocol.

### 2.21. Pharmacokinetics of Subcutaneously Delivered sMB08

Pharmacokinetic studies of sMB08 subcutaneous administration were performed in 10-week old rats. Three groups of rats were administered subcutaneously (SC) with sMB08 at different doses for each group. Blood samples were collected at different time points (0, 1, 4, 8, 24, 48, and 72 h) for a total of 72 h. Pharmacokinetic results were measured and analyzed using a calibrated ELISA detecting sMB08 levels.

### 2.22. Pharmacokinetics of Intranasaly Administered sMB08

Pharmacokinetic studies of sMB08 intranasal administration were evaluated in 11-weeks old mice. Six groups of mice were administered intranasaly (IN) with sMB08/Vehicle at different time points for each group. Pharmacokinetics results were measured and analyzed using a calibrated ELISA detecting sMB08 levels.

### 2.23. The Effect of sMB08 in the Rotenone Induced PD Model

The rotenone solution was first prepared as a 50× stock in 100% dimethylsulfoxide (DMSO) and diluted in olive oil, to obtain a final concentration of 1.5, 2, or 2.5 mg/mL rotenone in olive oil, 2% DMSO. Vortexing the solution creates a stable emulsion of the DMSO containing rotenone and olive oil. The solution was made fresh 2–3 times/week and stored in an amber septa vial protected from light and inverted several times before each injection to eliminate the possibility of settling. The solution was administered at 1 mL/kg, and control animals received the vehicle only. Experimental groups were comprised of at least 4–10 animals. Rotenone was administered by increased doses (day 0, day 2, and day 6) once a day for 12 consecutive days.

Twenty-four adult male (3 groups) Sprague-Dawley rats (175–200 g) were anesthetized with an intraperitoneal (i.p.) injection of ketamine hydrochloride (100 mg/kg) and xylazine (10 mg/kg), and injected daily i.p. for 12 days with 1.5 mg/kg/d Rotenone (dissolved in DMSO/Olive oil), 2 mg/kg/d from day 5 and 2.5 mg/kg/d from day 9 prepared fresh twice a week. Animals were euthanized when severe parkinsonian symptoms developed. Mortality occurring immediately after the injection (in the absence of parkinsonian symptoms and typically <10%) was excluded from analysis. Animals were treated by intranasal (i.n.) administration with 10 μg (~40 μg/kg) of sMB08 (5 μg in each nozzle) and PBS as sham at the first and every 3 days after (4 total). Behavior tests (Cylinder and Postural instability) were conducted at various predetermined time points before they were sacrificed at day 12. Transcardial perfusion with PBS and 4% formalin was performed in all animals. For histological studies, the brain was removed and underwent overnight postfixation with neutral buffered formalin (Thermo Fisher Scientific, Waltham, MA, USA), before being processed and embedded in O.C.T. For biochemical studies, tissues were immediately frozen after removal and stored at −80 °C until used. CSF was collected from all animals.

### 2.24. The Effect of sMB08 in a PD Mouse Model Inducible by αSyn PFF Stereotactic Injections

Fifty Male (5 groups of n = 10) C57BL/6JRccHsd mice (2–4 months of age) were anesthetized by Isoflurane and stereotactically injected with recombinant αSyn PFF (7 μg of PFF per brain) in PBS. Control C57BL/6JRccHsd animals received sterile PBS. A single needle insertion (coordinates: +0.2 mm relative to bregma, 2.0 mm from midline) into the right forebrain was used to target the inoculum to the dorsal neostriatum located at a depth of 2.6 mm below the dura. Material was injected via a 10-μL Hamilton syringe at a rate of 0.5 μL per min (3.5 μL total volume) with the needle in place (33G) for ≥10 min at each target. Animals were inoculated at the right hemisphere unless otherwise indicated. After recovery from surgery, animals were treated by intranasal (IN) administration with 3 concentrations of sMB08 (16 μg/kg, 80 μg/kg and 320 μg/kg) twice a week, and behavior tests (Grip test, Rota rod, Cylinder and NOR) were conducted at various predetermined time points before they were sacrificed at 9 weeks post injection by overdose with ketamine/xylazine and then transcardial perfusion with PBS (and additional perfusion with 4% formalin for histological studies). For histological studies, the brain was removed and underwent overnight postfixation with neutral buffered formalin (Thermo Fisher Scientific), before being processed and embedded in O.C.T compound (Ref 4583, Tissue-Tek)—used for binding tissue to the specimen block and to surround and cover the tissue specimen. For biochemical studies, tissues were immediately frozen after removal and stored at −80 °C until used.

### 2.25. Adeno Associated Virus (AAV) Preparation

AVV-αSyn construction: AAV2 backbone construct expressing the human alpha synuclein gene (SNCA) with AAV-DJ hybrid capsid (created from 8 different AAV serotypes) driven by the human CMV promoter. AAV construct vectors were cloned with a human alpha synuclein protein expression sequence through ClaI and XhoI enzymatic restriction sites. Viral production in HEK293 cells transfected with packaging plasmids (pHelper ΔS and RC-DJ) followed by cell lysis and heparin column purification.

### 2.26. The Effect of sMB08 in Rats with PD Inducible by Stererotactic AAV-αSyn Injection

Thirty eight female (5 groups) Sprague-Dawley rats (225–250 g) were anesthetized with an intraperitoneal injection of ketamine hydrochloride (100 mg/kg) and xylazine (10 mg/kg), and stereotactically injected with AAV–αSyn into the right hemisphere. Injections were made into the substantia nigra (SN) at the following coordinates: anteroposterior (AP), −5.3; mediolateral (ML), +1.7; dorsoventral (DV), −7.2;. Control animals received sterile PBS. Material was injected via a 10 μL Hamilton syringe at a rate of 0.3 μL per min with the needle in place (33G) for ≥10 min at each target. Animals were inoculated at the right hemisphere unless otherwise indicated. After recovery from surgery, animals were treated by intranasal (IN) administration with 3 concentrations of sMB08 (5 μg/kg, 50 μg/kg and 250 μg/kg) 2 times/week, behavior tests (Wire hang, Cylinder and Open Field) were conducted at various predetermined time points before they were sacrificed at 7 weeks post injections by overdose with ketamine/xylazine and then transcardial perfusion with PBS (and additional perfusion with 4% formalin for histological studies). For histological studies, the brain was removed and underwent overnight postfixation with neutral buffered formalin (Thermo Fisher Scientific, Waltham, Massachusetts, USA), before being processed and embedded in O.C.T. For biochemical studies, tissues were immediately frozen after removal and stored at −80 °C until used. CSF and blood serum were collected from all animals. The AAV2 vector was used to overexpress human wild-type αSyn, in which the expression of the transgene is driven by the human CMV promoter. A total of 8 × 10^8^ IFU (infectious units) were injected per animal (6 μL per animal).

### 2.27. Immunohistochemistry of Human and Murine Tissues

Brains were extracted and post-fixed in 4% PFA for 24 h and sunk in 30% sucrose. Brains were frozen on a microtome platform and cut to generate 16 and 40 μm thick sections. A series of free-floating coronal sections was stained for either tyrosine hydroxylase (TH) or phosphorylated αSyn (pSyn). Tissue was incubated in 0.3% H_2_O_2_ for 45 min, rinsed, and blocked in 10% normal goat serum (1 h), then incubated in primary mouse anti-pSyn (Ser129) (1:500, BioLegend, San Diego, CA, USA), mouse-anti-TH (1:200) antibodies overnight at 4 °C. Then, sections were incubated in biotinylated secondary antisera against either mouse (1:400, Millipore, Temecula, CA, USA) or rabbit IgG (1:400, Millipore, Temecula, CA, USA) followed by the Vector ABC detection kit (Vector Labs, Burlingame, CA, USA). Antibody labeling was visualized by exposure to 0.5 mg/mL 3,3′ diaminobenzidine (DAB), 2.5 mg/mL nickel ammonium sulfate and 0.03% H_2_O_2_ in Tris buffer followed by incubation with the NovaRed kit (Vector Labs, Burlingame, CA, USA). Sections were mounted on subbed slides, dehydrated to xylene, and coverslipped with xylene base mounting buffer.

## 3. Behavioral Assessment

### 3.1. Cylinder Test

Cylinder test is a motor assessment of forelimb asymmetry [21]. Rats were individually put into a glass cylinder (20 cm diameter, 34 cm height) and were video recorded for 5 min and until they touched the cylinder wall with their forelimbs 20 times. The recordings were analyzed by an investigator who was not aware of the identity of the rats. The data are presented as the following formula: (Left touch)/(Right touch + Left touch + Both touch).

### 3.2. Wire Hang Test

Neuromuscular strength was tested with a wire hang test [22]. The mice was placed on a wire mesh, waved gently so that the mouse gripped the wire, and then inverted. Latency to fall was recorded with a 15 min cut-off time. Each mouse was tested three times, and the top two results were used for analysis.

### 3.3. Rotarod Behavioral Analysis

The Ugo Basile 47,650—Mouse NG rotarod treadmill (Ugo Basile) was used to assess fine motor coordination and balance [22]. Rotarod performance is measured on a rotating rod as the mice must walk forward to avoid falling off a continuously rotating cylinder [23]. Pre-training consisted of 4 consecutive trials at low rotational speed of 20 rpm. Two trials were measured on a rod that accelerates from 0 to 50 rpm, and the average time on the rod for each mouse was used for data analysis. Time on the rod was used to assess fine motor coordination and balance.

### 3.4. Rearing Behavior

When placed in a clear cylinder, rats will engage in exploratory behavior, including rearing. During rearing behavior, the forelimbs will contact the wall of the cylinder.

For this test, the rat was placed in a clear plexiglass cylinder (height = 30 cm, diameter = 20 cm) for 3 min. The test was conducted under low red-light conditions and video-recorded, and during video playback, the number of rears was quantified. To be classified as a rear, the animal had to raise forelimbs above shoulder level and make contact with the cylinder wall with either one or both forelimbs. Removal of both forelimbs from the cylinder wall and contact with the table surface was required before another rear was scored. This test has been successfully used previously to assess behavioral deficits in the rats receiving subcutaneous or intravenous rotenone.

### 3.5. Novel Object Recognition (NOR)

Twenty-four hours before testing, all animals were habituated to the testing apparatus for 10 min (50 cm box, 40 cm high). The day after, animals were introduced to the objects: first, two identical objects were placed in the box, and mice were allowed to explore objects for 5 min. The same procedure continued until 5 mice were done. The entire phase for 5 mice lasted 30 min. Immediately after, these 5 mice were tested in the same order as before. Animals were introduced to two different objects, one familiar object and one novel object that the mice never encountered. Mice were allowed to explore objects for 5 min and then removed from the box. At all phases, after each mouse was removed from the box, the box was sterilized with alcohol. Sample and novel objects and their locations were counterbalanced across animals. Each trial was videotaped, and time and frequency spent with each object was measured using Noldus EthoVision XT 11.5 (Noldus information Technology, Wageningen, The Netherlands) [24].

### 3.6. Statistical Analysis

Values shown in the figures are presented as mean +/− SEM. P values for determination of the statistical significance of differences were calculated by means of paired, two-tailed Student’s *t* test, one-way ANOVA with a post hoc Dunnet’s, or one-way ANOVA with Tukey’s post test.

## 4. Results

### 4.1. sMB08 Exhibits a High Affinity to Both Human and Mouse αSyn PFF

**Figure 1:**
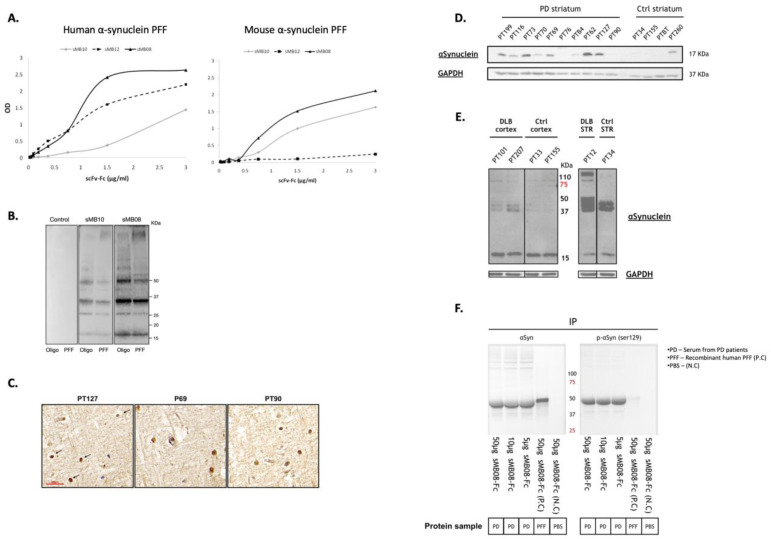
sMB08 selection, affinity and binding properties. (**A**) High affinity binding of scFv-Fc’s to human alpha synuclein PFF versus mouse alpha synuclein PFF in ELISA assay. ELISA results are in triplicates (technical sample repetition); (**B**) Western blots of scFv-Fc’s binding to human αSyn oligomers and PFF; (**C**) sMB08 recognizes αSyn Lewy bodies in the substantia nigra (PT127 and PT69) and cortex (PT90) of PD patients. Arrows indicate example of positive Lewy bodies. Data shown representative micrographs of substantia nigra and cortex sections from human brain of PD patients. Scale bar 30 µm; (**D**) Western blots of sMB08 binding to human striatum extracts from PD/control group patients; (**E**) sMB08 binding to human cortex (left panels) and striatum (STR—right panels) extracts of DLB and control group (Ctrl) patients. GAPDH as “housekeeping” protein loading control is shown in lower panels; (**F**) sMB08 (scFv-Fc for immunoprecipitation) immunoprecipitates αSyn from PD patients sera. Left panel—human αSyn staining using mouse monoclonal anti-human αSyn antibody (αSyn clone Syn 204); Right Panel—phosphorylated human αSyn staining using mouse monoclonal anti-human phosphorylated αSyn in serine 129 (P-αSyn (ser129)).

A standard ELISA was performed using human or mouse αSyn PFF as the coating protein (Figure 1A). sMB08-Fc, sMB10-Fc, and sMB12-Fc binds to both coating proteins in a concentration dependent manner (1:2 serial dilutions from 3 to 0.023 μg/mL). sMB08 showed the highest affinity to both human and mouse αSyn (Figure 1A; black line) compared to sMB10 (Figure 1A; grey line) and sMB12 (Figure 1A; dashed black line). sMB12 did not bind mouse protein.

### 4.2. sMB08 Binds αSyn Oligomers and PFF by WB

sMB08 bind both human alpha synuclein oligomers and PFFs with higher affinity than sMB10, as seen in the SDS-PAGE Western blot staining (Figure 1B). Control staining using an isotype control human IgG showed no staining.

### 4.3. sMB08 Staining of Alpha Synuclein Positive Lewy Bodies in Human PD Brains

sMB08 was detected alpha-synuclein Lewy neurites [6] in the substantia nigra (Figure 1C; PT127 and P69) and cortex (Figure 1C; PT90) of patients with PD as seen in the immunohistochemical staining of Figure 1C.

### 4.4. sMB08 Binds Tissues from Patients with PD/DLB by WB

sMB08 (scFv) detected αSyn in several human brain (striatum, cortex) PD, dementia with Lewy bodies (DLB), and control extracts seen in SDS-PAGE Western blot staining (Figure 1D,E), demonstrating its ability to recognize modified forms of αSyn in multiple patients with synucleinopathies.

### 4.5. sMB08-Fc Immunoprecipitates αSyn from PD Patients’ Sera

The main component of pathological lesions (almost 90%) in patients with PD is extensively phosphorylated at αSyn serine 129 (pS129 αSyn). Phosphorylation at Ser129 can regulate αSyn fibril formation and enhance αSyn toxicity both in vitro and in vivo. Although trace levels of phosphorylated αSyn are detectable in normal brains, nearly all αSyn accumulated within Lewy bodies in PD brains is phosphorylated on serine 129 (Ser-129). Phosphorylated αSyn can be secreted from neuronal cells and can be seen in the serum and CSF of PD patients. Human sera from PD patients were immunoprecipitated with sMB08-Fc to determine its ability to bind endogenous human alpha-synuclein. Three different concentrations of sMB08-Fc (50 µg, 10 µg, and 5 µg) immunoprecipitated αSyn from PD patients’ sera (Figure 1F). Samples were stained with a commercial anti-human αSyn (Figure 1F, left panel) and anti-human phosphorylated (Ser-129) αSyn (Figure 1E, right panel). Human recombinant PFF immunoprecipitation with sMB08-Fc served as positive control for αSyn binding (Figure 1F, left panel) and negative control for serine 129 αSyn phosphorylation (there is no phosphorylation in serine 129, seen in Figure 1F, right panel).

### 4.6. sMB08 Protects Human Neuronal-like Cells from αSyn PFF/Oligomer and PD Brain Extract Induced Toxicity

**Figure 2:**
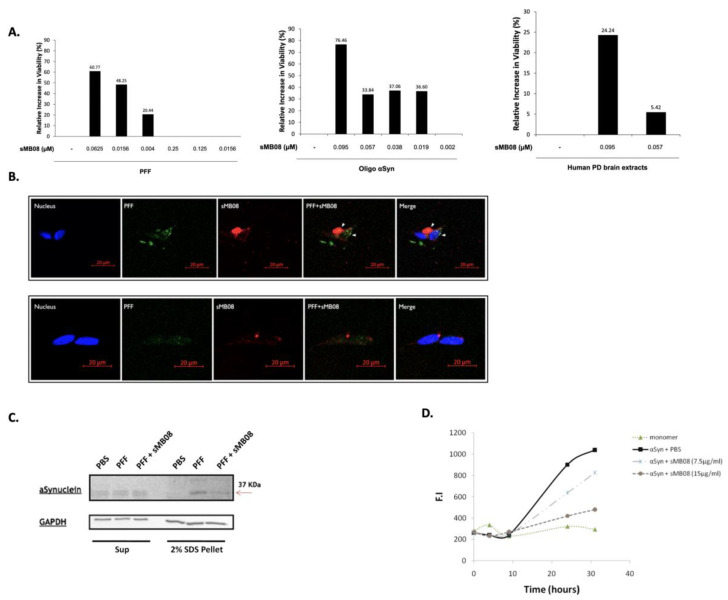
sMB08 penetrates neurons and attenuates aggregation and spreading of αSyn. (**A**) Bar graphs showing neuronal increase in viability mediated by sMB08 (scFv). sMB08 protects neuronal SH-SY5Y cells from αSyn PFF (left), oligomers (middle), and human PD patient brain extracts (right) mediated toxicity. The *y*-axis represents the relative increase in cell viability compared to PFF/oligomers/human PD brain extracts with no treatment (PBS), sMB08 treatment, and 80R scFv isotype treatment (sham); (**B**) sMB08 penetration to human neuronal cells. Confocal images of intracellular co-staining of αSyn PFF conjugated to Alexa 488 (green) and sMB08 conjugated to Cy5 (red). DAPI was used for nuclear staining in cells (blue). Scale bar is 20 µm; (**C**) Western blot following sequential extraction of neurons in 1% (*v*/*v*) TX-100 followed by SDS. Immunoblotting was performed using rabbit anti-rodent αSyn, an antibody that recognizes endogenous total αSyn (upper panel) and mouse anti-GAPDH (lower panel). In PFF-treated neurons, there was an increase in SDS-soluble αSyn (2% SDS) compared to PBS or PFF+sMB08 treated neurons (red arrow); (**D**) Kinetics for the formation of β-sheet-rich assemblies: human αSyn incubated with sMB08 (7.5 μg/mL or 15 μg/mL) versus PBS (black solid line). Monomers (dotted line marked with triangles) were measured as baseline.

The cytotoxicity of αSyn PFF and oligomers was assessed on differentiated human neuroblastoma SH-SY5Y cells using the 3-(4,5-dimethylthiazol-2-yl)-2,5-diphenyltetrazolium bromide (MTT) assay (Figure 2A). αSyn PFF (Figure 2A; left graph) and αSyn oligomers (Figure 2A; middle graph) had a toxic effect on cells in low concentrations as seen by the cell viability assay (Figure 2A). Incubation of PFF αSyn in the presence of increasing concentrations of sMB08 improved cell viability in a dose dependent manner, while no affect was seen in the presence of sham (80R) (Figure 2A; left graph). Incubation of αSyn oligomers with increasing concentrations of sMB08 improved cell viability at the concentration of 10 µg/mL (~76%) with a similarly moderate increase in 6 µg/mL, 4 µg/mL, and 1 µg/mL concentrations of sMB08 (~35%). A sMB08 concentration of 0.2 µg/mL had no effect in cell viability (Figure 2A; middle graph). Human PD brain extracts (1:1000 in PBS) had a toxic cellular effect compared to control extracts (data not shown). sMB08 was protected cells in a dose dependent manner (~24% and ~5%) as evidenced by increasing cell viability as seen in Figure 2A (right graph).

### 4.7. sMB08 Penetrates Neurons and Co-Locolizes with αSyn PFF

sMB08 penetration to neurons cells was assessed on differentiated human neuroblastoma SH-SY5Y cells using confocal microscopy for detection. sMB08 conjugated to Cy5 was visualized intracellular in neuronal cells co-stained with αSyn PFF conjugated to Alexa 488, as seen in merge confocal images (Figure 2B; PFF + sMB08).

### 4.8. sMB08 Abrogates Spreading and Seeding of Human PFF In Vitro

In this cell-based model, the ability of sMB08 to abrogate PFF seeding was tested. Small seeds of PFF generated from recombinant αSyn were added directly to primary neurons with and without sMB08. These seeds of PFFs induced recruitment of endogenously expressed αSyn into abnormal, phosphorylated, insoluble and ubiquinated aggregates. The formation of these aggregates from endogenous αSyn in primary neurons derived from wild-type, nontransgenic mice follows an initial lag phase of 2–4 days, which are then seen in the SDS extract of neuronal cells (insoluble protein) in the nitrocellulose membrane immunostaining (Figure 2C; upper panel—2% SDS Pellet). sMB08 was able to abrogate endogenous αSyn aggregation by inhibiting the human PFF prion-like recruitment (Figure 2C; Red arrow; PFF + sMB08-upper panel). Higher levels of murine alpha-synuclein are seen the SDS extract after human PFF “seeding” without sMB08 (Figure 2C; PFF-upper panel). To confirm that the increase in mis αSyn in the neurons is the result of endogenous corruption and not externally uptaken protein, the antibody used for detection was anti-murine αSyn, not cross reactive with human.

### 4.9. sMB08 Inhibits αSyn Aggregation In Vitro

Aggregation of misfolded proteins is believed to occur when hydrophobic residues exposed at the surface of proteins interact with other misfolded proteins. Human recombinant αSyn monomers can be driven to aggregate by constant shaking (orbital shaker) 1000 rpm at 37 °C for 7 days (Figure 2D). As seen in Figure 2D, sMB08 (αSyn + sMB08 7.5 µg/mL and αSyn + sMB08 15 µg/mL) has the ability to inhibit αSyn aggregation (measured by Thioflavin T aggregated protein staining assay), whereas αSyn without sMB08 (αSyn + PBS) aggregates. αSyn monomers were measured at each time point for reference (monomer).

### 4.10. sMB08 Attenuates Cellular Uptake of αSyn Oligomers and PFF in Human PBMCs and Mouse Microglia

**Figure 3:**
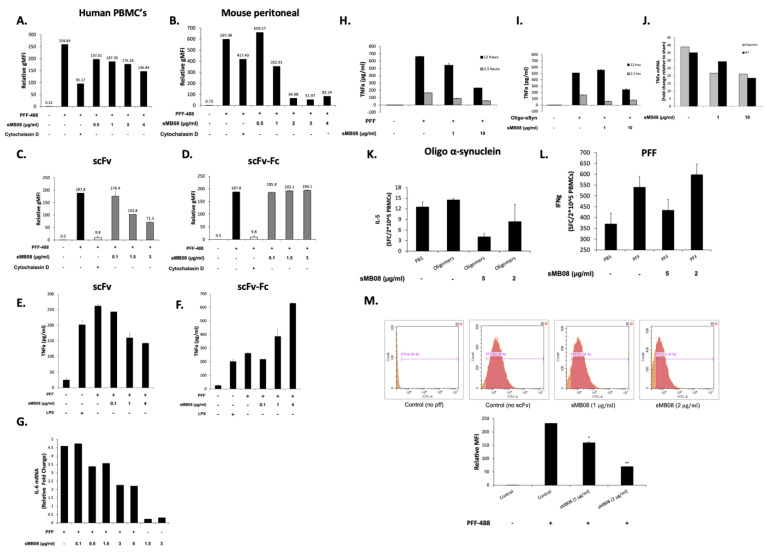
sMB08 ameliorates neuroinflammation and facilitates misolded-αSyn degradation (**A**) sMB08 attenuates uptake in a dose dependent manner of fluorescent PFF in human PBMCs. The *y*-axis represents the relative geometric mean (gMFI) measured by flow cytometry; (**B**) sMB08 attenuates uptake in a dose dependent manner of fluorescent PFF in mouse peritoneal macrophages. The *y*-axis represents the relative geometric mean (gMFI) measured by flow cytometry; sMB08 (**C**) attenuates uptake a dose dependent manner of fluorescent PFF compared to sMB08-Fc (**D**) in microglia cells (BV2); (**E**) sMB08 reduces TNFα protein levels in mouse microglia BV-2 cells compared to (**F**) sMB08-Fc elevates TNFα protein levels. (**G**) sMB08 attenuates IL-6 mRNA levels in mouse microglia BV-2 cells. Real-time PCR was done in cell homogenates. The *y*-axis represents the fold-change in expression compared to no treatment (cells only). GAPDH was used as “housekeeping” gene; sMB08 attenuates inflammatory cytokines in human PBMCs induced by αSyn PFF and oligomers. PBMCs were activated with PFF (**H**) and oligomers (**I**) for 2.5 h or 12 h to and TNFα was measured (**J**) sMB08 attenuates inflammation in human PBMCs induced by αSyn PFF and oligomers. PBMCs were activated with PFF (**K**) and oligomers (**L**) for 2.5 h to measure TNFα at the mRNA expression levels. Real-time PCR was done in cell homogenates. The *y*-axis represents the fold-change in expression compared to no treatment (cells only). GAPDH was used as “housekeeping” gene; (**M**) sMB08 lowers fluorescent PFF levels in a dose dependent manner in neuronal cells (SH-SY5Y). The *y*-axis represents the relative geometric mean (gMFI) measured by flow cytometry. ** *p* < 0.002, * *p* < 0.05.

One of the main functions of macrophages and microglia in the immune system is phagocytosis, facilitating the clearance of debris. They can also serve as antigen presenting cells that promote T cell mediated cellular immunity. Flow cytometry was used to measure PFF/oligomers conjugated to Alexa fluor 488 (PFF-488/Oligo-488) cellular uptake (calculated by relative geomean fluorescent intensity (gMFI)) (Figure 3A–D). Flow cytometry analysis shows that sMB08 in scFv format attenuates human and mouse peritoneal macrophages cellular uptake of PFF conjugated to Alexa fluor 488 compared to cells with PBS treatment seen in Figure 3A,B respectively. sMB08 in scFv format (Figure 3C) attenuates mouse microglia cellular uptake of PFF conjugated to Alexa fluor 488 in a dose dependent manner, whereas Fc containing antibodies do not (Figure 3D).

### 4.11. sMB08 Attenuates Neuroinflammation Induced by αSyn Oligomers and PFF in Human PBMCs and Mouse Microglia

Microglia is one of the major cell types involved in the inflammatory responses in the central nervous system (CNS) that appear to contribute to neuroinflammation, evident from the expression of tumor necrosis factor-α (TNF-α; TNFa), interleukin-1β (IL-1β), and interferon-gamma (IFN-γ) in the midbrain of PD patients which strongly suggest the involvement of immune components in PD pathogenesis. The scFv form of sMB08 down-regulates (in a dose dependent manner) pro-inflammatory cytokines (TNF-α, IL-6) produced by microglia after exposure to PFF both (Figure 3E,G), whereas Fc containing anti-αSyn antibodies increase the expression of these cytokines (Figure 3F).

sMB08 down-regulates (in a dose dependent manner) TNF-α produced by human PMBC after PFF (Figure 3H) or oligomers αSyn (Figure 3I) exposure at both 2.5 and 12 h. Furthermore, sMB08 down-regulates TNF-α mRNA levels after PFF and oligomers αSyn exposure (Figure 3J; 1 µg/mL and 10 µg/mL).

### 4.12. sMB08 Attenuates Neuroinflammation by Tuning Down the Autoimmune Response of T Cells to αSyn Oligomers and PFF 

Alpha synuclein aggregated forms act as antigenic epitopes and drive helper and cytotoxic T cell responses in patients with PD. To determine whether αSyn PFF/oligomers were recognized by T cells, HLA class I type, and HLA class II responses were assayed. IFNγ was used as a representative cytokine to detect CD8^+^ HLA class I and CD4^+^ T helper 1 (Th1) class II T cells, and IL-5 was used as a representative cytokine secreted by CD4^+^ T helper 2 (Th2) class II T cells.

Fourteen days of αSyn oligomers PBMCs stimulation compared to un-stimulated PBMCs (PBS) increased the number of IL-5 secreted cells when re-exposed to αSyn oligomers (Figure 3K), indicating a prominent CD4^+^ Th2 phenotype. The addition of sMB08 (5 µg/mL and 2 µg/mL) to αSyn oligomers managed to tune down by half the autoimmune response as seen in Figure 3K. PBMCs response to the αSyn PFF after 14 days of PFF stimulation increased twice the number of IFNγ secreted cells (Figure 3L) compared to un-stimulated PBMCs (PBS), indicating a prominent CD8^+^ HLA class I and CD4^+^ T helper 1 (Th1) class II T cells phenotype. The addition of sMB08 (5 µg/mL) to αSyn PFF tuned down the immune response.

### 4.13. sMB08 Facilitates Degradation of Aggregated PFF in Human Neuroblastoma Cells

To determine whether sMB08 has protective effects in differentiated neuroblastoma cells treated with αSyn PFFs, SH-SY5Y cells were treated first with PFF conjugated to Alexa Fluor 488 and 16 h later exposed to sMB08 for 48 h. Using flow cytometry, PFF degradation was seen in neuronal cells treated with sMB08 in a dose-dependent manner (Figure 3M; 1 µg/mL—159.41 ± 4.02 gMFI, 2 µg/mL—6.25 ± 1.09 gMFI) compared to control cells with no sMB08 treatment (Figure 3M; 231.85 ± 0.95 gMFI).

### 4.14. sMB08 Attenuates Neuroinflammation In Vivo in Murine Cortex of Animals Injected with Alpha Synuclein Oligomers and PFF

**Figure 4:**
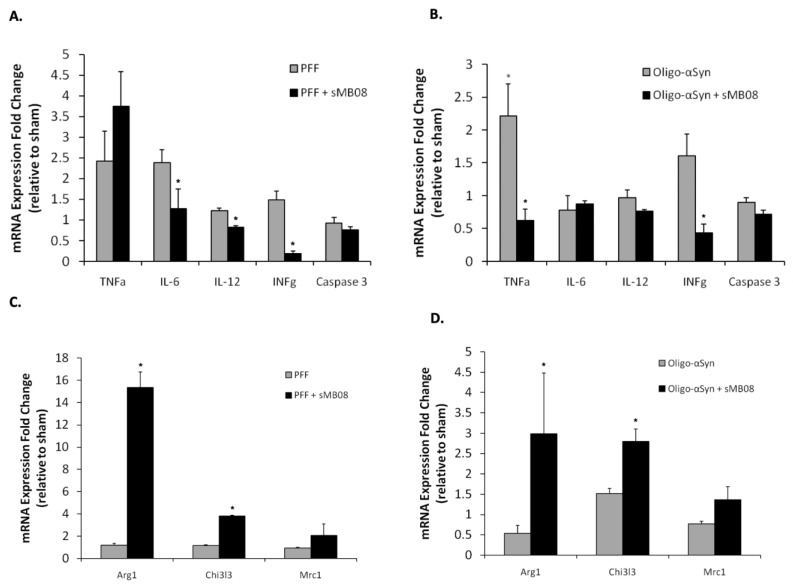
The effect of sMB08 on neuroinflammation in mice stererotactically injected with mis aSyn. Graphs showing that sMB08 attenuates neuroinflammation in murine cortex of animals injected (striatum) with PFF (**A**,**C**) and oligomers (**B**,**D**). Real-time PCR was done in brain cortical homogenates. The expression of TNFa, IL-6, IL-12, INFγ, Caspase 3, Arg1, Chi3l3, and Mrc1 was determined by TaqMan real-time PCR in brain homogenates. The *y*-axis represents the fold-change in expression after PFF intrastriatal injections (with and without sMB08 treatment). GAPDH was used as “housekeeping” gene. * *p* < 0.05. Error bars represent standard error of the mean.

Mice were injected with human αSyn PFF into the striatum (with and without sMB08 treatment) to examine in vivo neuroinflammation. The scFv form of sMB08 down-regulated a panel of pro-inflammatory cytokines and up-regulated anti-inflammatory genes produced in mice brain after 72 h of exposure to human PFF (Figure 4A,C) or oligomers (Figure 4B,D) αSyn. Figure 4A shows that sMB08 significantly down-regulates IL-6, IL-12, and INFγ pro-inflammatory cytokines mRNA expression levels after PFF exposure. Figure 4B shows that sMB08 significantly down-regulates TNFa and INFγ pro-inflammatory cytokines mRNA expression levels after oligomers exposure, whereas Figure 4C shows that sMB08 significantly up-regulates Arg1 (Arginase 1) and Chi3l3 (Ym1) mRNA expression levels after PFF exposure. Figure 4D shows that sMB08 significantly up-regulates Arg1 and Chi3l3 mRNA expression levels after oligomers exposure.

### 4.15. Intranasal Administration of sMB08 Attenuates Motor Dysfunction Induced by Alpha Synuclein Aggregation—Rotenone in Rats

**Figure 5:**
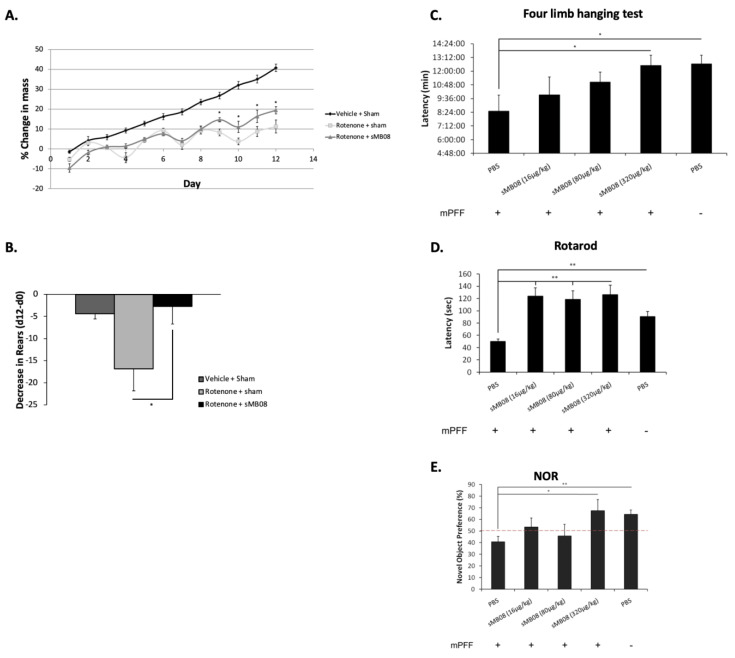
sMB08 treatment in the rotenone induced PD model. (**A**) Change of mass in rats injected with rotenone (Rotenone + Sham, n = 10), rotenone with sMB08 treatment (Rotenone + sMB08, n = 10), and control rats (Vehicle + Sham, n = 4). * *p* < 0.05. Error bars represent standard error of the mean. (**B**) Quantification of number of rears in rats. * *p* < 0.05. Error bars represent standard error of the mean. Mouse alpha-synuclein PFF induced PD. (**C**) Neuromuscular strength assessment using a wire hang test. * *p* < 0.05. Error bars represent standard error of the mean. (**D**) Rotarod test performed at 10 weeks after injection. ** *p* < 0.002. Error bars represent standard error of the mean. (**E**) Mean novel object preference for all mice groups during the testing phase when one familiar object and one novel object were presented to mice is shown. ** *p* < 0.002, * *p* < 0.05. Error bars represent standard error of the mean.

In the rotenone model, systemic inhibition of mitochondrial complex I produces selective degeneration of the nigrostriatal dopamine system and reproduces key pathological features of clinical PD. Indeed, rotenone administration affects many of the pathogenic pathways including: oxidative stress, αSyn phosphorylation and aggregation and Lewy pathology, DJ-1 acidification and translocation, proteasomal dysfunction and nigral iron accumulation. Daily intraperitoneal injections of rotenone in natural oil (olive oil) were given for 12 days in 20 animals. Four animals were injected with natural oil only as a control group. Animals were treated by IN administration of sMB08 (50 µg/kg) and PBS as sham at day 1, 4, 7, and 10. Animal weight was measured each day in order to calculate % change in mass (Figure 5A). sMB08 IN treatment was able to significantly reduce loss of weight as shown in Figure 5A (Rotenone + sMB08) at days 9–12. Animals were placed in a transparent cylinder to quantify rearing behavior (forelimbs contact with the wall of the cylinder). Animals treated IN with sMB08 exhibited a decrease in rearing behavior (Figure 5B; rotenone + sMB08) as compared with the sham treated animals (Figure 5B; Rotenone + sham) at day 12 compared to day 0.

### 4.16. sMB08 Treatment Attenuates Motor Dysfunction in the Mouse PFF IC PD Model

Sonicated fibrils made from recombinant α-synuclein, when added to primary cultured neurons or injected into the striatum or other brain areas, can produce robust formation of inclusions that resemble Lewy bodies and Lewy neurites found in diseased brains. Thus, this model of PFF injection allows for investigation of the impact of misfolded αSyn on the neuronal function and consequent phenotypes, and determination of whether preventing inclusion formation can reverse these phenotypes. Inclusion formation occurs in neurons from hippocampus, cortex, and midbrain and other brain regions, and thus the impact of these aggregates on diverse neuronal populations can be examined. Regardless of the injection site, αSyn inclusions form at the injection site and appear in brain areas distant from the site of injection. In this model, defects occur well before any neuron death, suggesting that neuronal dysfunction emerges in response to abnormal α-synuclein very early before neurodegeneration begins. As shown in Figure 5C,D, mice were evaluated for their latency to fall after a number of motor tests eight weeks post intra-striatal mouse αSyn PFF injection. A wire hang test was used to test neuromuscular strength. Animals injected with mouse PFF and IN treated with PBS (Figure 5C; +mouse PFF + PBS; ~8:30 min) had significantly lower latency than animals injected with PBS and treated with PBS used as control (Figure 5C; −mouse PFF + PBS; ~12:00 min). sMB08 IN treated animals showed a significant increase in their latency to fall (+mouse PFF + sMB08 (320 µg/kg); ~12:00 min) as compared to untreated animals (+mouse PFF + PBS; ~8:30 min). Rotarod treadmill was used to assess fine motor coordination and balance in treated mice. Three different concentrations of sMB08 (Figure 5D; 16 µg/kg, 80 µg/kg and 320 µg/kg) increased murine latency to fall (Figure 5D; + mouse PFF + sMB08; ~120 s) as compared to untreated animals with mouse PFF intra-striatal injections (Figure 5D; + mouse PFF + PBS; ~50 s). Figure 5E shows the cognitive decline of mouse PFF injected animals (Figure 5E; + mouse PFF + PBS) as compared to PBS injected controls (Figure 5E; −mouse PFF + PBS), as measured by their preference to a novel object. Above 50% (Figure 5E), animal preference is for the novel object (less cognitive decline). Intranasal treatment with sMB08 (Figure 5E; + mouse PFF + sMB08 (320 µg/kg); ~65%) significantly improved cognitive decline as compared to untreated animals (Figure 5E; +mouse PFF + PBS; ~45%). The experiment was conducted in 50 animals, 10 animals per group (n = 10).

### 4.17. sMB08 Intranasal Treatment Attenuates Motor Dysfunction Induced by Intracerebral Injections of AAV-αSyn in Rats

**Figure 6:**
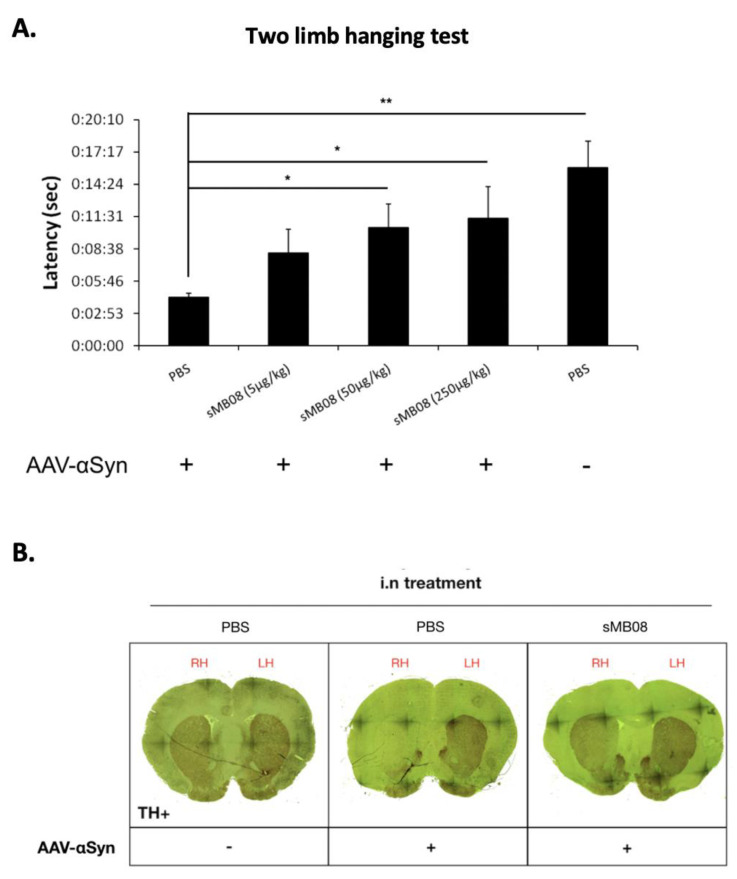
Treatment of sMB08 in the AAV-αSyn induced PD rat model. (**A**) Neuromuscular strength assessment using a two limb wire hang test. * *p* < 0.05, ** *p* < 0.01. Error bars represent standard error of the mean; (**B**) Brain sections of animals injected with AAV-αSyn that develop extensive and significantly greater loss of TH^+^ and dopaminergic (DA) neurons and the effect of sMB08.

One of the advantages of the rAAV-α-synuclein model is that αSyn can be expressed in dopaminergic neurons of the SNpc for long periods of time (a cell population particularly vulnerable in PD). Misfolding of αSyn leads to a progressive loss of dopaminergic neurons in the SNpc, and loss of dopamine terminals in the striatum with significant defects in motor behavior. The rapid, early loss of striatal dopamine function seen in the rAAV-αSyn model replicates the pattern seen in human disease. Furthermore, neuroinflammation is one of the most robust phenotypes found by rAAV-αSyn over-expression, and replicates many features seen in human PD. Testing neuromuscular strength, Figure 6A shows, 6–8 weeks post intra-striatal injections of rAAV-αSyn in rats, a reduction in their latency to fall (Figure 6A; +AAV-αSyn + PBS; ~3.5 s) in a two limb hanging test as compared to sham injected animals (Figure 6A; −AAV-αSyn + PBS; ~15 s). Intranasal repeated treatments of sMB08 (twice a week) in rats post intra-striatal injections of rAAV-αSyn were able to attenuate motor dysfunction by significantly increasing the animals’ latency to fall (Figure 6A; +AAV-αSyn + sMB08 (50 µg/kg) and (250 µg/kg); ~9:00 s and ~10 s respectively).

### 4.18. sMB08 Protects against Dopaminergic (DA) Neuron Loss

Figure 6B shows that animals injected with adeno-associated virus expressing human αSyn (AAV-αSyn) develop extensive loss of TH^+^ neurons in SN pars compacta and striatum 6 weeks post injections as seen in tyrosine hydroxylase immunostaining of brain coronal slices (Figure 6B—middle panel; right hemisphere (RH)). sMB08 given IN (twice a week) decreases loss of TH^+^ nigral neurons induced by AAV-αSyn in rats (Figure 6B—right panel). There is no loss of TH^+^ neurons seen in sham injected animals six weeks post injections (Figure 6B—left panel).

### 4.19. Subcutaneous Administration of sMB08 in Rats-Pharmacokinetics

**Figure 7:**
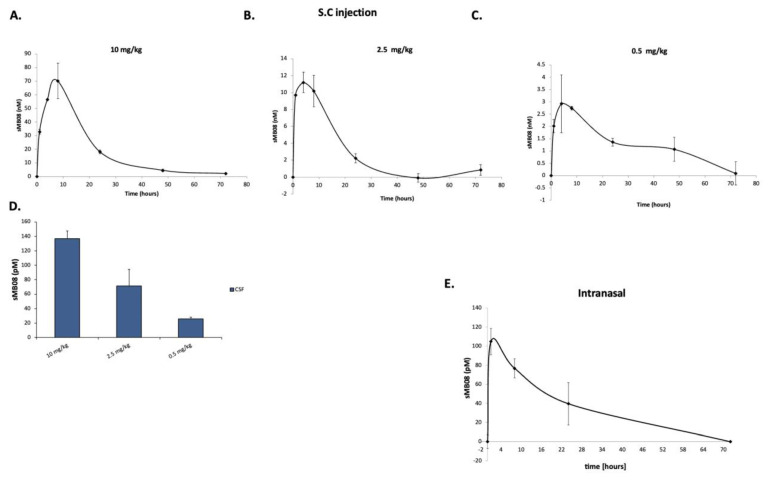
Pharmacokinetic analysis of sera and CSF in treated rats—sMB08 levels in rat serum in three doses of SC treatment: 10 mg/kg (**A**), 2.5 mg/kg (**B**) and 0.5 mg/kg (**C**). sMB08 levels in rats CSF 24 h post treatment (**D**). Error bars represent standard deviation of the mean. (**E**) sMB08 levels in murine brains. Error bars represent standard deviation of the mean.

sMB08 levels in serum and CSF samples from rats were detected using a calibrated ELISA. The ELISA was able to detect sMB08 levels in CSF of rats in all three doses of treatment (Figure 7A–C) even 72 h post treatment. The half-life of sMB08 was 16 h, calculated from the two highest doses analyzed curves (Figure 7A,B). Calculated CSF/Serum ratio was 1.04% (Figure 7).

### 4.20. IN Administration of sMB08 in Mice-Pharmacokinetics

sMB08 levels in brain samples from mice were detected using a calibrated ELISA. The ELISA was able to detect sMB08 levels in mice 1, 8, and 24 h post intranasal treatment (Figure 7E).

## 5. Discussion

The pathogenesis of synucleinopathies in general and of PD in specific, is complex and multifactorial and no disease modifying approach has proven successful in pivotal clinical trials. The optimal therapeutic agent would need to address the different triggering mechanisms that likely synergize to result in neurodegeneration of dopaminergic neurons. Herein, we aimed at exploring an agent that was designed to overcome the limitations of current biologic agents while addressing each of the factors involved in the pathogenesis of PD.

Misfolded forms of αSyn play a critical role in mediating dopaminergic nerve loss and would thus stand as a valid target despite the uncertainty with regard to the importance of oligomeric forms versus PFFs. We performed a comprehensive screening using human Fab phage display libraries so as to select the optimal lead that was cloned to the scFv format exhibiting a high affinity to both αSyn oligomers and PFF. The scFv exhibited negligible cross reactivity to the native αSyn monomer. sMB08 exhibited a high potency in achieving a dose dependent inhibition of in vitro αSyn aggregation. In parallel, we have been able to document a consistent protective effect of sMB08 on neuron-like cells from both oligomeric and PFF αSyn inducible toxicity. This observation is corroborated by a similar protective effect of sMB08 that was evident when toxicity to the neurons was triggered by extracts of brains from PD patients. These sMB08 properties are likely to play a role in the potentially protective effect of this scFv in the in vivo models of PD, although therapeutic approaches that address only these mechanisms, including mAbs and aggregation inhibitors, failed to achieve meaningful benefit in clinical trials [6].

The direct toxicity of misfolded αSyn to neurons is further boosted by spreading and seeding of the pathology. Indeed, mounting evidence exists to support of a chain of event that begins after exteriorization of misfolded αSyn following cell death, that is uptaken by surrounding neurons [6]. These misfolded uptaken αSyn forms seed and corrupt endogenous αSyn, thus driving a propagated and templated process that results in spreading of the pathology over neuronal niches. We have been able to show that sMB08 is capable of inhibiting the process of seeding and spreading in vitro. This may be related to its ability to protect against misfolded αSyn mediated neuronal toxicity. In this respect, a specific and unique property of sMB08 relates to its ability to passively penetrate the neuron and colocalize with cytosolic misfolded αSyn as shown in the confocal studies. This finding may suggest that sMB08 engagement can also provide neuroprotection by blocking the *intracellular* corruption of endogenous αSyn by internalized misfolded forms of the protein. The advantage of scFv fragments in this respect is obvious: whereas full length mAbs cannot penetrate the cell due to their high molecular weight, scFv that are sixth the size of mAbs, are capable of passively traversing cell membrane to potentially engage the toxic misfolded forms of αSyn. This ability is likely to be therapeutically meaningful as the vast majority of misfolded αSyn pool resides in the intracellular space and thus not accessible to full length mAbs [6]. Moreover, we have been able to show that this passive neuronal entry by sMB08 is associated with degradation of labeled misfolded αSyn. This observation is consistent with a previous study where a scFv targeting TDP-43 mediated ubiquination and degradation of misfolded TDP43 [25].

Neuroinflammation, driven both by the innate and the adaptive immune system activation, appears to play an important role in the pathogenesis of PD [4]. This was exemplified by a series of studies showing evidence for microglial activation and the presence of activated T cells in the brains of patients with PD [4]. Moreover, proinflammatory cytokines produced by these activated immune cell players are overexpressed in PD brains so as to further strengthen the role of neuroinflammation in driving and perpetuating neurodegeneration. Accordingly, recent data also suggest that T cell lines generated from patients with PD target misfolded epitopes of αSyn and exhibit a proinflammatory phenotype upon recall stimulation with this misfolded protein [26]. This observation is complimentary to studies attesting to the indirect role of T cells expressed in PD brains [27]. We have made a serial assessment of sMB08, one of the relevant innate and adaptive immune cells, aiming to predict its activity as an antigen specific neuroinflammatory agent. We have shown that the uptake of both αSyn oligomers and PFF to microglia is attenuated by sMB08 and consequently the secretion of proinflammatory agents by these innate immune cells is tuned down. Interestingly, when we tested sMB08 fused to an IgG Fc portion, the anti-inflammatory effect was lost, suggesting that protection from inflammation afforded by full length IgG mediated uptake of misfolded αSyn could be counteracted by non-specific Fc mediated activation of fc gamma receptors acting to promote cytokine release. The lack of the Fc portion in the scFv, which still retains the ability to block uptake of misfolded αSyn without generating an Fc mediated non-specific inflammatory activation, stands as a clear advantage over using a classic mAb.

The lack of the Fc portion in the scFv format and the ability to block internalization of misfolded forms of αSyn to microglia serving as antigen presenting cells led us to test the hypothesis that sMB08 may act as an antigen specific inhibitor of adaptive αSyn reactive T cells [26,27]. We followed the data generated by Sulzer et al. [26] showing evidence of T-cell lines from PD patients reactive with misfolded αSyn by testing the hypothesis that sMB08 can interfere with αSyn mediated T cell activation. Indeed, we have shown that αSyn specific T cell lines from PD patients, restimulated with mis-αSyn in the presence of sMB08 exhibited a reduced activation status evident by attenuated IFN-gamma and IL-5 secretion by ELISPOT. We next investigated whether the in vitro effects of sMB08 are also evident in vivo. For this purpose, we tested the effects of sMB08 on oligomer and PFF mediated neuroinflammation after intrastriatal delivery to murine brains. Indeed, treatment with sMB08 was associated with a significant attenuation of the proinflammatory program consequent to misfolded αSyn intracerebral instillation and to a shift in microglial phenotype towards an anti-inflammatory M2 like signature.

One of the major hurdles in using biologic agents in CNS disorders is the lack of effective BBB penetration that prevents adequate delivery of these relatively large proteins. In this respect, the much smaller scFv would also have an advantage over full length mAbs. However, the obvious drawback would be their shorter half-life, due to the lack of the stabilizing Fc, thus making the delivery of these agents challenging due to the need for frequent dosing. We have thus explored the possibility of using sMB08 via the intranasal route that provides a more pragmatic method of accomplishing frequent dosing. Several studies have tested the feasibility of attaining therapeutic dosing in CNS disorders. The major advantage of this method apart from the ease of use, is that ability to bypass the BBB by advancing the agent via paraneural routes [28,29]. Studies showing proof of concept for the ability to achieve meaningful dosing have been obtained not only for small molecules, but also for small sized proteins, such as albumin and insulin. We have made a series of pharmacokinetic studies showing that achievement of therapeutic doses of sMB08 is attainable via the intranasal route and these studies were also designed to select the dosing schedule and frequency by determining the half-life of the scFv. As there is no optimal model of experimental PD, we tested the efficacy of sMB08 in three different models capturing different pathomechanistic aspects of the disease, namely: neurotoxicity, spreading and seeding, and neuroinflamation. Employing the rotenone, AAV-αSyn, and the PFF models, we have been able to show repeatedly that intranasal treatment with sMB08 resulted in a significant protection from motoric dysfunction. This was accompanied by a considerable preservation of the hypocampal dopaminergic nerves evidenced by the TH+ immunohistochemical studies. The consistency with regard to the efficacy of sMB08 in the different PD models attest to the wide spectrum of protective activities observed in our in vitro assays as these models exhibit motoric dysfunction resulting from various mechanistic triggers.

## 6. Conclusions

The relative failures that were evident in the clinical trials employing αSyn mAbs in the clinic are in sharp contrast to the consistently efficacious results obtained with these same agents in preclinical trials. Whereas such inconsistencies may result from the lack of appropriate modeling of the human disease by these murine models, they could also result from inherent problems of using full length mAbs. These are very large molecules containing an Fc portion with nonspecific inflammatory properties. We selected, cloned, and tested a scFv that has the ability to overcome some of the drawbacks of IgG while preserving and enhancing some of its beneficial attributes. Accordingly, the relatively small size of sMB08 allows for an effective intranasal delivery and neuronal penetration, culminating in the engagement of mis-αSyn and neuroprotection, while avoidance of the Fc portion disposes of the ‘off target’ immune activation effects. Thus, sMB08 may stand as a novel approach of treating PD patients if acceptable bioavailability is obtained in humans by the intranasal route.

## 7. Patents

The results are partially reported in a filed patent.

## Data Availability

Can be provided by the corresponding author.
